# Supplementary material for: Spatial transcriptomics reveals antiparasitic targets associated with essential behaviors in the human parasite Brugia malayi
Source: PLoS Pathog. 2022 Apr 7;18(4):e1010399. doi: 10.1371/journal.ppat.1010399 (PMC9017939; doi:10.1371/journal.ppat.1010399)
Supplement: S1 File — A list of anatomical description references, utilized for comparative analyses shown in Fig 2A, garnered from the available literature for filarial parasites related to Brugia malayi. (PDF) [file ppat.1010399.s005.pdf]

Supplementary File 1: Comparative anatomy references of Clade III nematodes

Key references denoting relative locations of key structures in Clade III nematodes selected by phylogenetic closeness to *Brugia malayi* according to previous publications.[1,2]

Species include:
Brugia malayi [3–7], Brugia timori [8–10], Brugia pahangi [6,11–14], Wuchereria bancrofti [5,15–19], Loa loa [5,20,21], Litomosoides petteri [22], Acanthocheilonema odendhali [23–25], Elaeophora schneideri [26,27], Mansonella interstitium [28], Mansonella perstans [29], Dirofilaria immitis [5,30,31], Onchocerca volvulus [32–34], Oxyspirura conjunctivalis [35], Oxyspirura mansoni [36], Gongylonema pulchrum [37,38], Dracunculus globocephalus [39,40], Dracunculus lutrae [41], Anisakis simplex [42–44], Ascaris lumbricoides [45], Ascaris suum [46,47], Parascaris equorum [48], Toxocara canis [49–52], Enterobius anthropopitheci [53], Enterobius vermicularis [54–56], and *Lemuricola nycticebi [57].*

References:

1. Smythe AB, Holovachov O, Kocot KM. Improved phylogenomic sampling of free-living nematodes enhances resolution of higher-level nematode phylogeny. BMC Evol Biol. 2019;19: 121.

2. Consortium IHG, International Helminth Genomes Consortium. Comparative genomics of the major parasitic worms. doi:10.1101/236539

3. Seo BS. Morphology of the microfilaria of Brugia malayi in Cheju-Do, Korea. Kisaengchunghak Chapchi. 1976;14: 41–49.

4. Mutafchiev Y, Bain O, Williams Z, McCall JW, Michalski ML. Intraperitoneal development of the filarial nematode Brugia malayi in the Mongolian jird (Meriones unguiculatus). Parasitol Res. 2014;113: 1827–1835.

5. Taylor AE. Studies on the microfilariae of Loa loa, Wuchereria bancrofti, Brugia malayi, Dirofilaria immitis, D. repens and D. aethiops. J Helminthol. 1960;34: 13–26.

6. Buckley JJC, Edeson JFB. On the Adult Morphology of Wuchereria sp. (malayi?) from a Monkey (Macaca irus) and from Cats in Malaya, and on Wuchereria pahangi n.sp. from a Dog and a Cat. Journal of Helminthology. 1956. pp. 1–20. doi:10.1017/s0022149x00032922

7. Vincent AL, Ash LR, Frommes SP. The Ultrastructure of Adult Brugia malayi (Brug, 1927) (Nematoda: Filarioidea). J Parasitol. 1975;61: 499.

8. Purnomo, Purnomo, Dennis DT, Partono F. The Microfilaria of Brugia timori (Partono et al. 1977 = Timor Microfilaria, David and Edeson, 1964): Morphologic Description with Comparison to Brugia malayi of Indonesia. J Parasitol. 1977;63: 1001.

9. Partono F, Dennis DT, Atmosoedjono S, Oemijati S, Cross JH. Brugia timori sp. n. (nematoda: filarioidea) from Flores Island, Indonesia. J Parasitol. 1977;63: 540–546.

10. David HL, Edeson JF. Filariasis in Portuguese Timor, with observations on a new microfilaria found in man. Ann Trop Med Parasitol. 1965;59: 193–204.

11. Schacher JF. Morphology of the microfilaria of Brugia pahangi and of the larval stages in the mosquito. J Parasitol. 1962; 679–692.

12. Schacher JF. Developmental stages of Brugia pahangi in the final host. J Parasitol.

1962;48: 693–706.

13. Vincent AL, Portaro JK, Ash LR. A Comparison of the Body Wall Ultrastructure of Brugia pahangi with that of Brugia malayi. J Parasitol. 1975;61: 567–570.

14. Collin WK. Ultrastructural morphology of the esophageal region of the infective larva of Brugia pahangi (nematoda: Filarioidea). J Parasitol. 1971;57: 449–468.

15. Chitwood BG, Chitwood MB, Others. An introduction to nematology. Section I. Anatomy. Monumental Printing Company, Baltimore; 1950.

16. Bain O. Recherches sur la morphogénèse des Filaires chez l'hôte intermédiaire. Ann Parasitol Hum Comp. 1972;47: 251–303.

17. Bain O, Dissanaike AS, Cross JH, Harinasuta C, Sucharit S. Morphologie de Wuchereria bancrofti adulte et sub-adulte. - Recherche de caractères différentiels entre les souches. Ann Parasitol Hum Comp. 1985;60: 613–630.

18. Schacher JF, Geddawi MK. An analysis of speciation and evolution in Wuchereria bancrofti by the study of nuclear constancy (eutely) in microfilariae. Ann Trop Med Parasitol. 1969;63: 67–82.

19. Araujo AC, Figueredo-Silva J, Souto-Padrón T, Dreyer G, Norões J, De Souza W. Scanning electron microscopy of adult Wuchereria bancrofti (Nematoda: Filarioidea). J Parasitol. 1995;81: 468–474.

20. Williams P. Studies on Ethiopian Chrysops as possible vectors of loiasis. II. Chrysops silacea Austen and human loiasis. Ann Trop Med Parasitol. 1961;55: 1–17 contd.

21. Eberhard ML, Orihel TC. Development and larval morphology of Loa loa in experimental primate hosts. J Parasitol. 1981;67: 556–564.

22. Bain O, Petit G, Berteaux S. Description de deux nouvelles Filaires du genre Litomosoides et de leurs stades infestants. Ann Parasitol Hum Comp. 1980;55: 225–237.

23. Perry ML, Forrester DJ. Dipetalonema odendhali (Nematoda: Filarioidea) from the northern fur seal, with a description of the microfilaria. J Parasitol. 1971;57: 469–472.

24. Kuzmina TA, Kuzmin YI, Tkach VV, Spraker TR, Lyons ET. Ecological, morphological, and molecular studies of Acanthocheilonema odendhali (Nematoda: Filarioidea) in northern fur seals (Callorhinus ursinus) on St. Paul Island, Alaska. Parasitol Res. 2013;112: 3091–3100.

25. Perry ML. A new species of Dipetalonema from the California sea lion and a report of Microfilariae from a Steller sea lion (Nematoda: Filarioidea). J Parasitol. 1967;53: 1076–1081.

26. Hibler CP, Adcock JL. Redescription of Elaeophora Schneideri Wehr and Dikmans, 1935 (Nematoda: Filarioidea). J Parasitol. 1968;54: 1095–1098.

27. Hibler CP, Metzger CJ. Morphology of the larval stages of Elaeophora schneideri in the intermediate host and definitive host with some observations on their pathogenesis in abnormal definitive hosts. J Wildl Dis. 1974;10: 361–369.

28. Price DL, Others. Description of Dipetalonema interstitium n. sp. from the grey squirrel and Dipetalonema llewellyni n. sp. from the raccoon. Proc Helminthol Soc Wash.

1962;29: 77–82.

29. Chabaud AG. Le genre Dipetalonema diesing 1861 ; essai de classification. Ann Parasitol Hum Comp. 1952;27: 250–294.

30. Taylor AE. The development of Dirofilaria immitis in the mosquito Aedes aegypti. J Helminthol. 1960;34: 27–38.

31. Orihel TC. Morphology of the larval stages of Dirofilaria immitis in the dog. J Parasitol. 1961;47: 251–262.

32. Strote G, Bonow I. Ultrastructure study of the excretory system and the genital primordium of the infective stage of Onchocerca volvulus (Nematoda:Filarioidea). Parasitol Res. 1995;81: 403–411.

33. Bain O. Redescription de cinq espèces d'Onchocerques. Ann Parasitol Hum Comp. 1975;50: 763–788.

34. Bain O. Morphologie des stades larvaires d'Onchocerca volvulus chez Simulium damnosum et redescription de la microfilaire. Ann Parasitol Hum Comp. 1969;44: 69–81.

35. Ivanova E, Spiridonov S, Bain O. Ocular oxyspirurosis of primates in zoos: intermediate host, worm morphology, and probable origin of the infection in the Moscow zoo. Parasite. 2007;14: 287–298.

36. Schwabe CW. Studies on Oxyspirura mansoni, the tropical eyeworm of poultry. II. Life history. 1951. Available: https://scholarspace.manoa.hawaii.edu/bitstream/10125/8798/vol5n1-18-35.pdf

37. Alicata JE. Early Developmental Stages of Nematodes Occurring in Swine. U.S. Department of Agriculture; 1935.

38. Kudo N, Kuratomi K, Hatada N, Ikadai H, Oyamada T. Further observations on the development of Gongylonema pulchrum in rabbits. J Parasitol. 2005;91: 750–755.

39. Moravec F, Little MD. Redescription of Dracunculus globocephalus Mackin, 1927 (Nematoda: Dracunculidae), a parasite of the snapping turtle, Chelydra serpentina. Folia Parasitol . 2004;51: 339–345.

40. Mackin JG. Dracunculus globocephalus n. sp., from Chelydra serpentina. J Parasitol. 1927;14: 91–94.

41. Crichton VFJ, Beverley-Burton M. Dracunculus lutrae n. sp. (Nematoda: Dracunculoidea) from the otter, Lutra canadensis, in Ontario, Canada. Can J Zool. 1973;51: 521–529.

42. Grabda J. Studies on the life cycle and morphogenesis of Anisakis simplex (Rudolphi, 1809) (Nematoda: Anisakidae) cultured in vitro. Acta Ichthyol Pisc. 1976;06: 119–141.

43. Ishii Y, Fujino T, Weerasooriya MV. Morphology of Anisakine Larvae. In: Ishikura H, Namiki M, editors. Gastric Anisakiasis in Japan: Epidemiology, Diagnosis, Treatment. Tokyo: Springer Japan; 1989. pp. 19–29.

44. Smith JW. Anisakis simplex (Rudolphi, 1809, det. Krabbe, 1878) (Nematoda: Ascaridoidea): morphology and morphometry of larvae from euphausiids and fish, and a review of the life-history and ecology. J Helminthol. 1983;57: 205–224.

45. Nichols RL. The Etiology of Visceral Larva Migrans: II. Comparative Larval Morphology of Ascaris lumbricoides, Necator americanus, Strongyloides stercoralis and Ancylostoma caninum. J Parasitol. 1956;42: 363.

46. Jenkins DC. The ultrastructure of the “excretory” system of ascaris suum larvae. Zeitschrift für Parasitenkunde. 1971;36: 179–192.

47. Fagerholm HP, Nansen P, Roepstorff A, Frandsen F, Eriksen L. Growth and structural features of the adult stage of Ascaris suum (Nematoda, Ascaridoidea) from experimentally infected domestic pigs. J Parasitol. 1998;84: 269–277.

48. Pilitt PA, Lichtenfels JR, Madden PA. Differentiation of Fourth and Early Fifth Stages of Parascaris equorum (Goeze, 1782) Nematoda: Ascaridoidea. 1979 [cited 2 Apr 2021]. Available: https://digitalcommons.unl.edu/parasitologyfacpubs/842/

49. Schacher JF. A contribution to the life history and larval morphology of Toxocara canis. J Parasitol. 1957;43: 599–610 passim.

50. Sprent JF. Observations on the development of Toxocara canis (Werner, 1782) in the dog. Parasitology. 1958;48: 184–209.

51. Vegni-Talluri M, Dallai R. Ultrastructure of the excretory-secretory system in Toxocara canis (Nematoda, Ascarididae) infective larvae. Boll Zool. 1989;56: 285–290.

52. Nichols RL. The etiology of visceral larva migrans. I. Diagnostic morphology of infective second-stage Toxocara larvae. J Parasitol. 1956;42: 349–362.

53. Hasegawa H, Ikeda Y, Fujisaki A, Moscovice LR, Petrzelkova KJ, Kaur T, et al. Morphology of chimpanzee pinworms, Enterobius (Enterobius) anthropopitheci (Nematoda: Oxyuridae), collected from chimpanzees, pan troglodytes, on Rubondo island, Tanzania. J Parasitol. 2005;91: 1314–1317.

54. Hulinska D, Others. The development of the female Enterobius vermicularis and the morphogenesis of its sexual organ. Folia Parasitol . 1968; 15–27.

55. Hugot JP, Tourte-Schaefer C. Étude morphologique des deux Oxyures parasites de l’homme :Enterobius vermicularisetE. gregorii. Ann Parasitol Hum Comp. 1985;60: 57–64.

56. Philpot F. Notes on the eggs and early development of some species of Oxyuridæ. J Helminthol. 1924;2: 239–252.

57. Frias L, Hasegawa H, Stark DJ, Lynn MS, Nathan SK, Chua TH, et al. A pinworm’s tale: The evolutionary history of Lemuricola (Protenterobius) nycticebi. Int J Parasitol Parasites Wildl. 2019;8: 25–32.
