# Supplementary figures and images for: Spatial transcriptomics reveals antiparasitic targets associated with essential behaviors in the human parasite Brugia malayi
Source: PLoS Pathog. 2022 Apr 7;18(4):e1010399. doi: 10.1371/journal.ppat.1010399 (PMC9017939; doi:10.1371/journal.ppat.1010399)

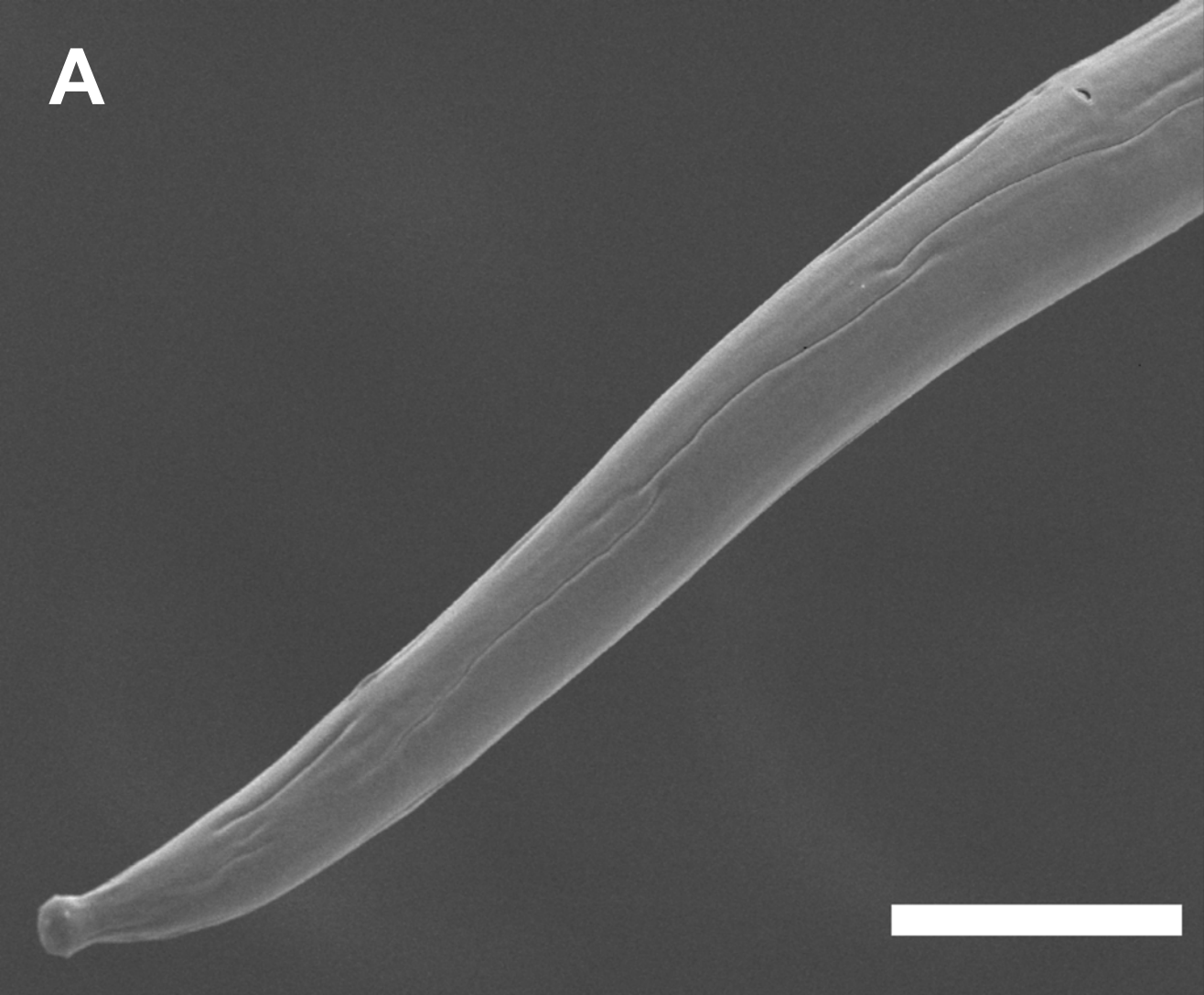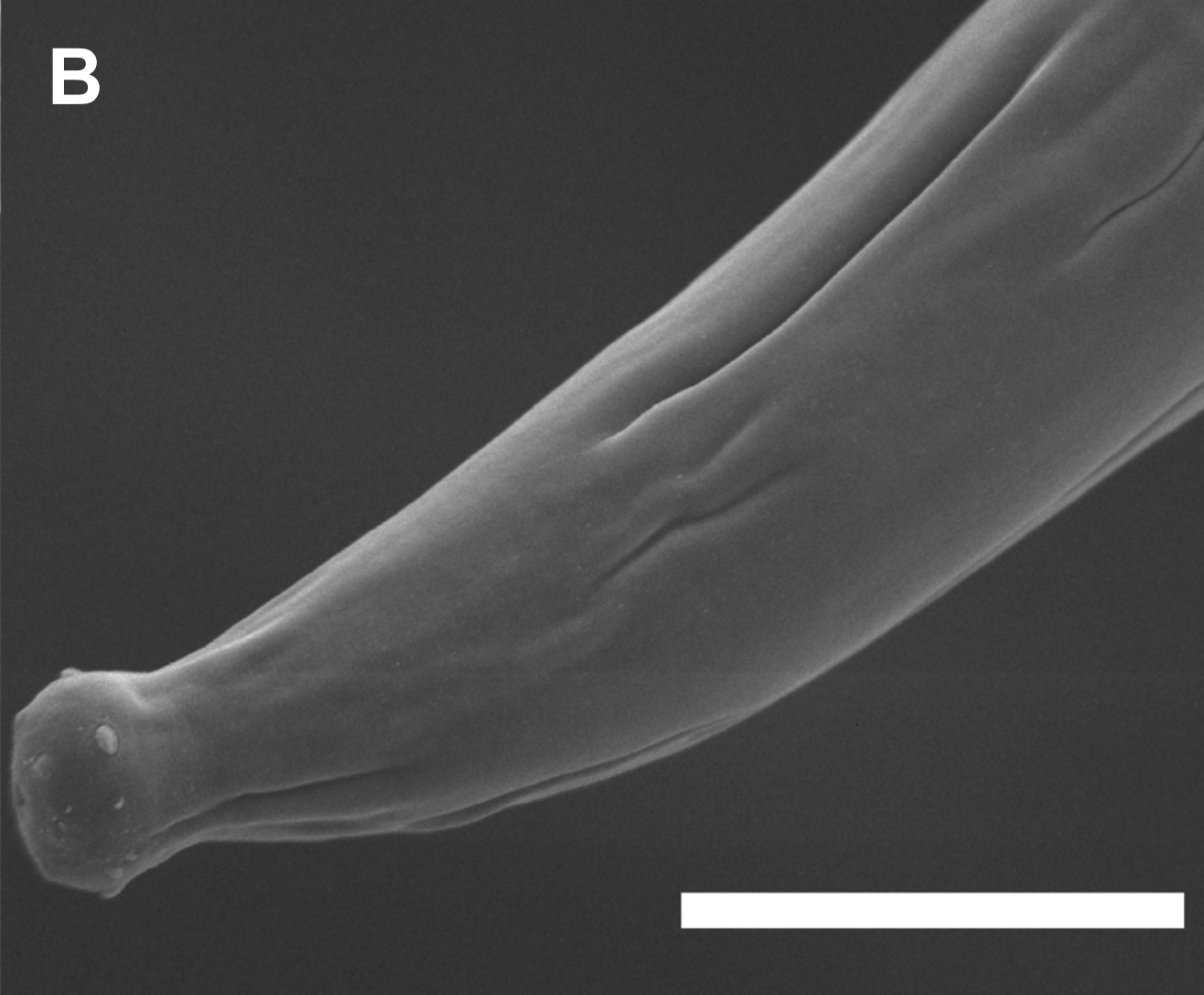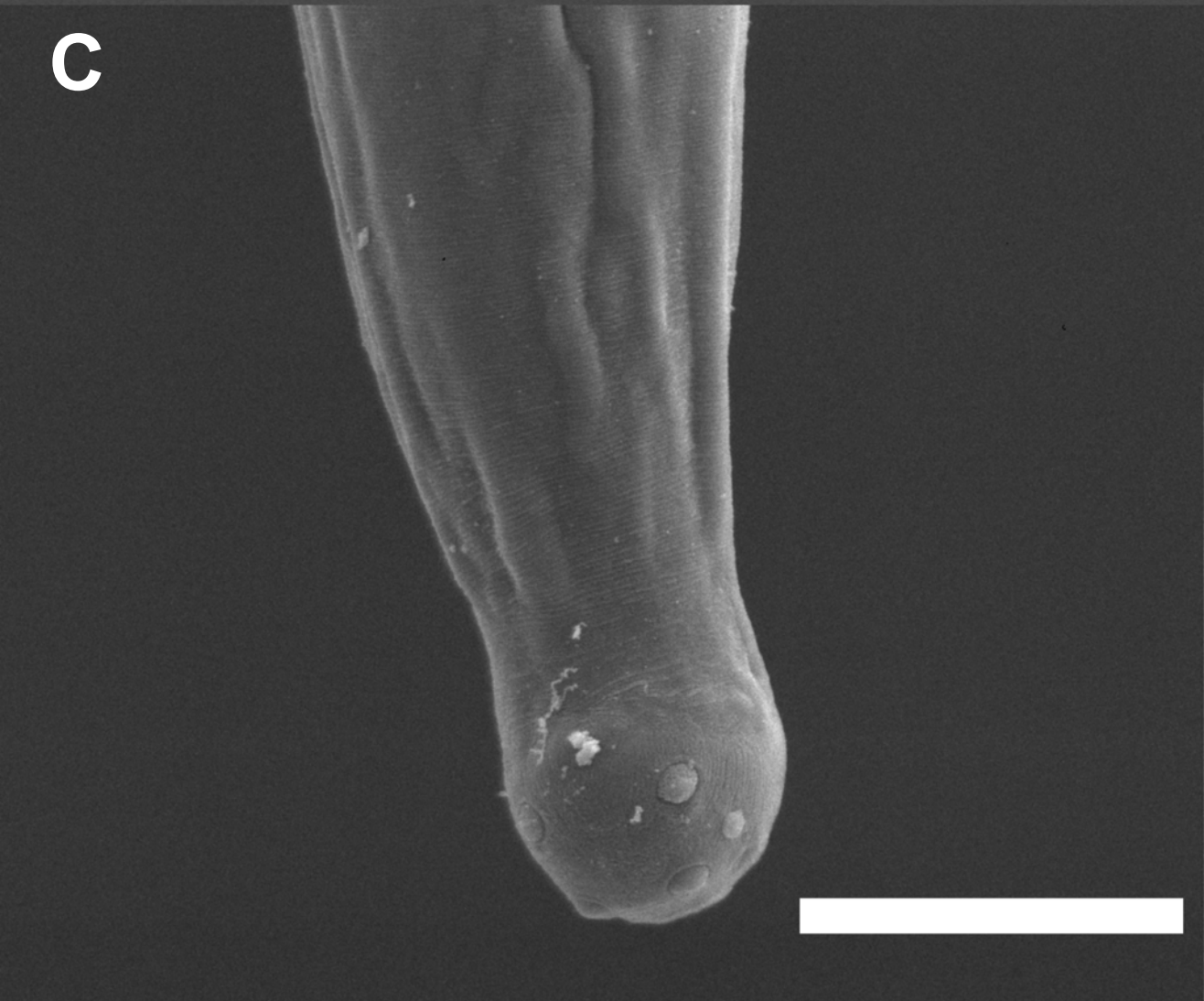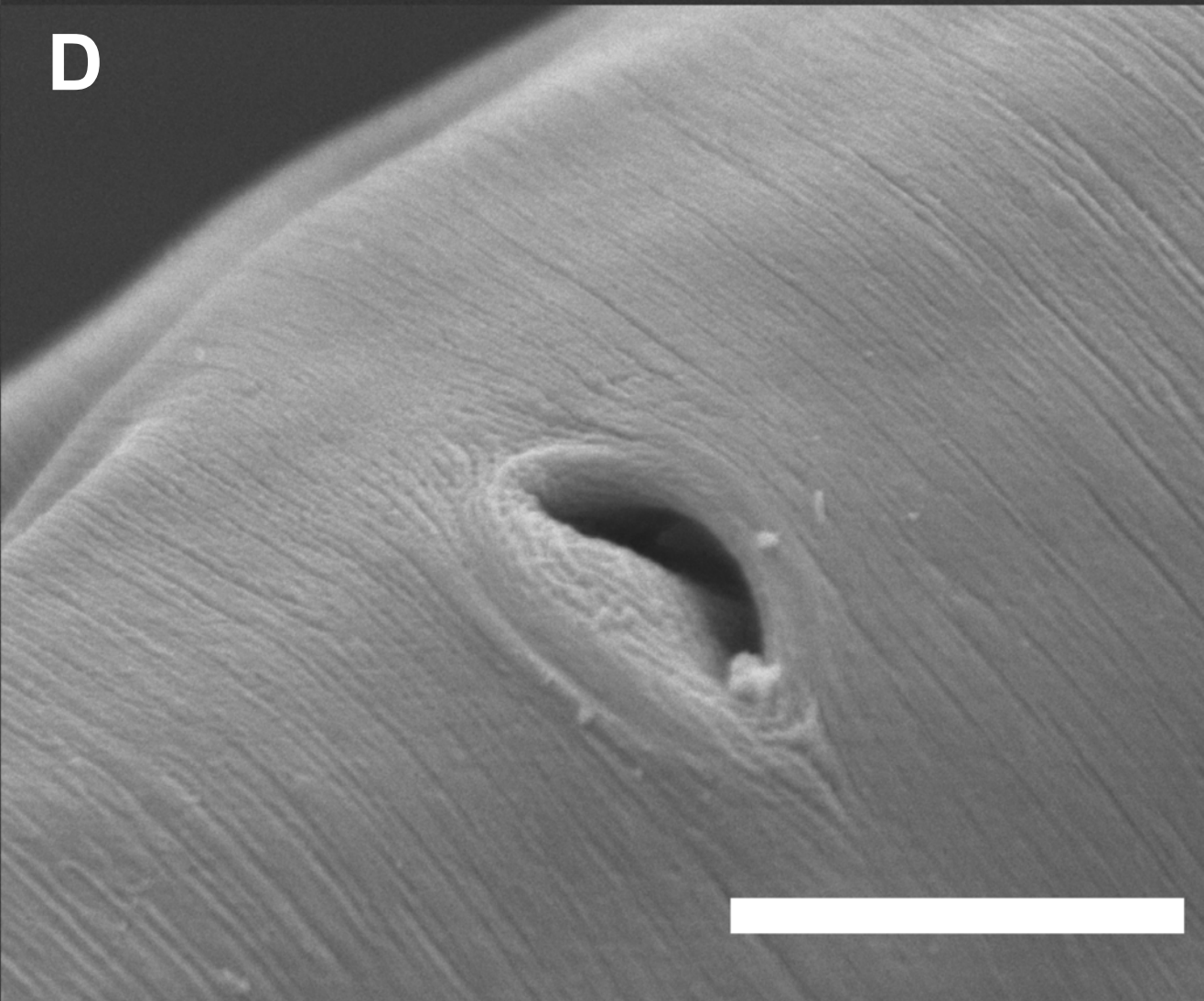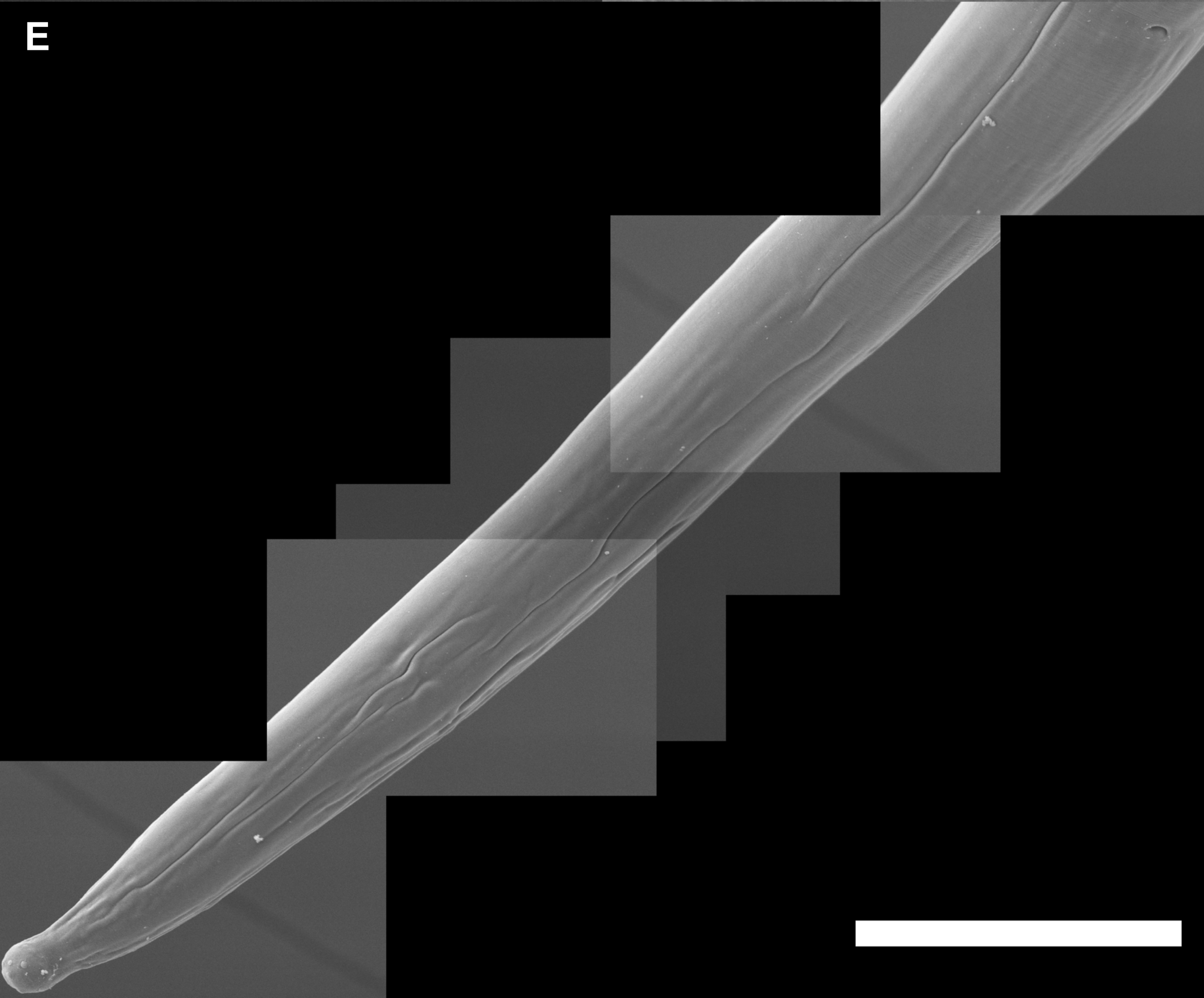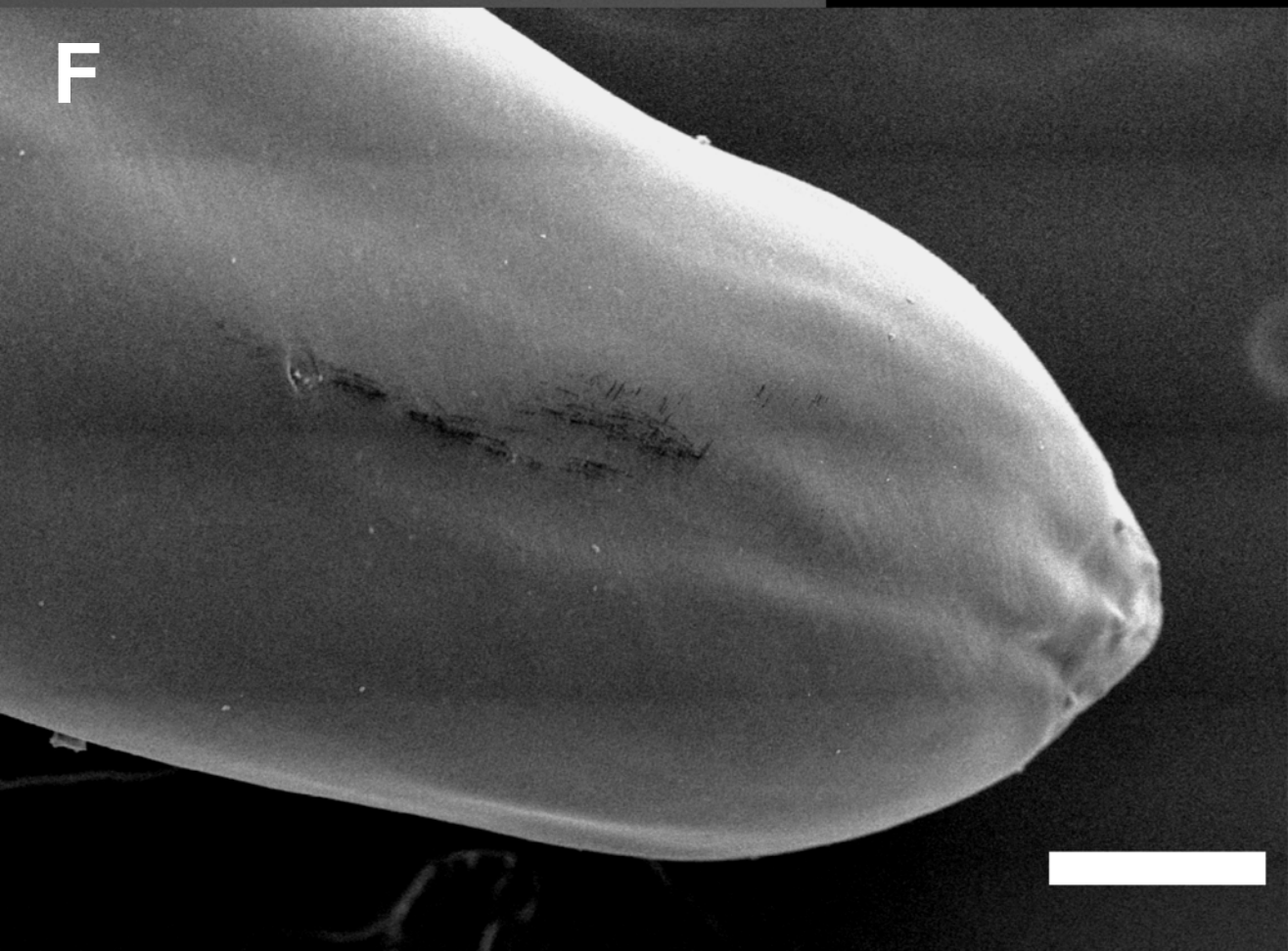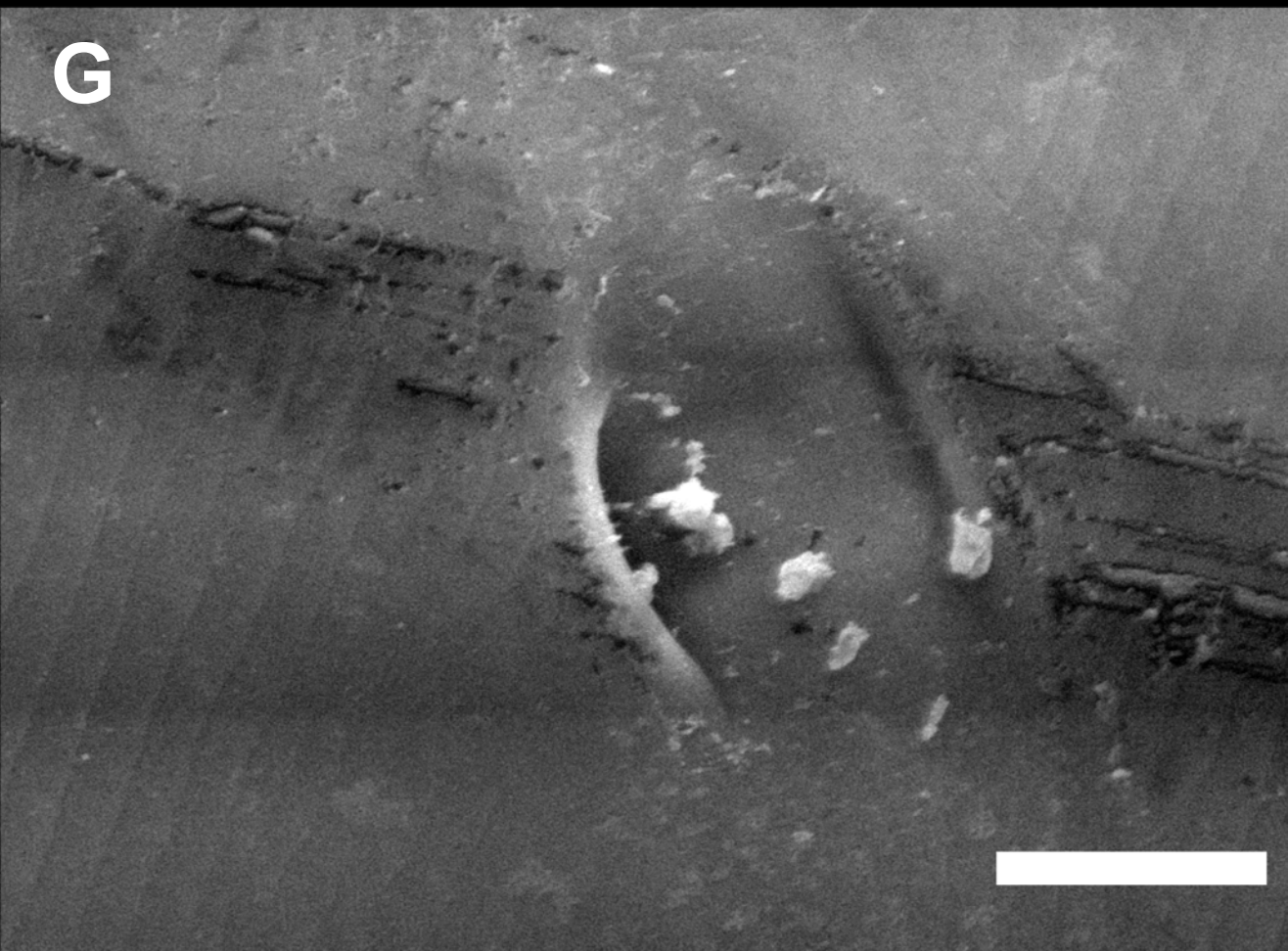

Supplement: S1 Fig — (A-E) Brugia pahangi. (A) Anterior to vulva single plane, scale = 100 μm, (B) head zoom from A, scale = 50 μm, (C) en-face view, scale = 25 μm, (D) close up of vulva, scale = 10 μm, (E) multi-focus merge from head to vulva, scale = 100 μm. (F-G) Dirofilaria immitis (F) head including vulva, scale = 100 μm and (G) close up of vulva, scale = 10 μm. (PDF) [file ppat.1010399.s007.pdf]

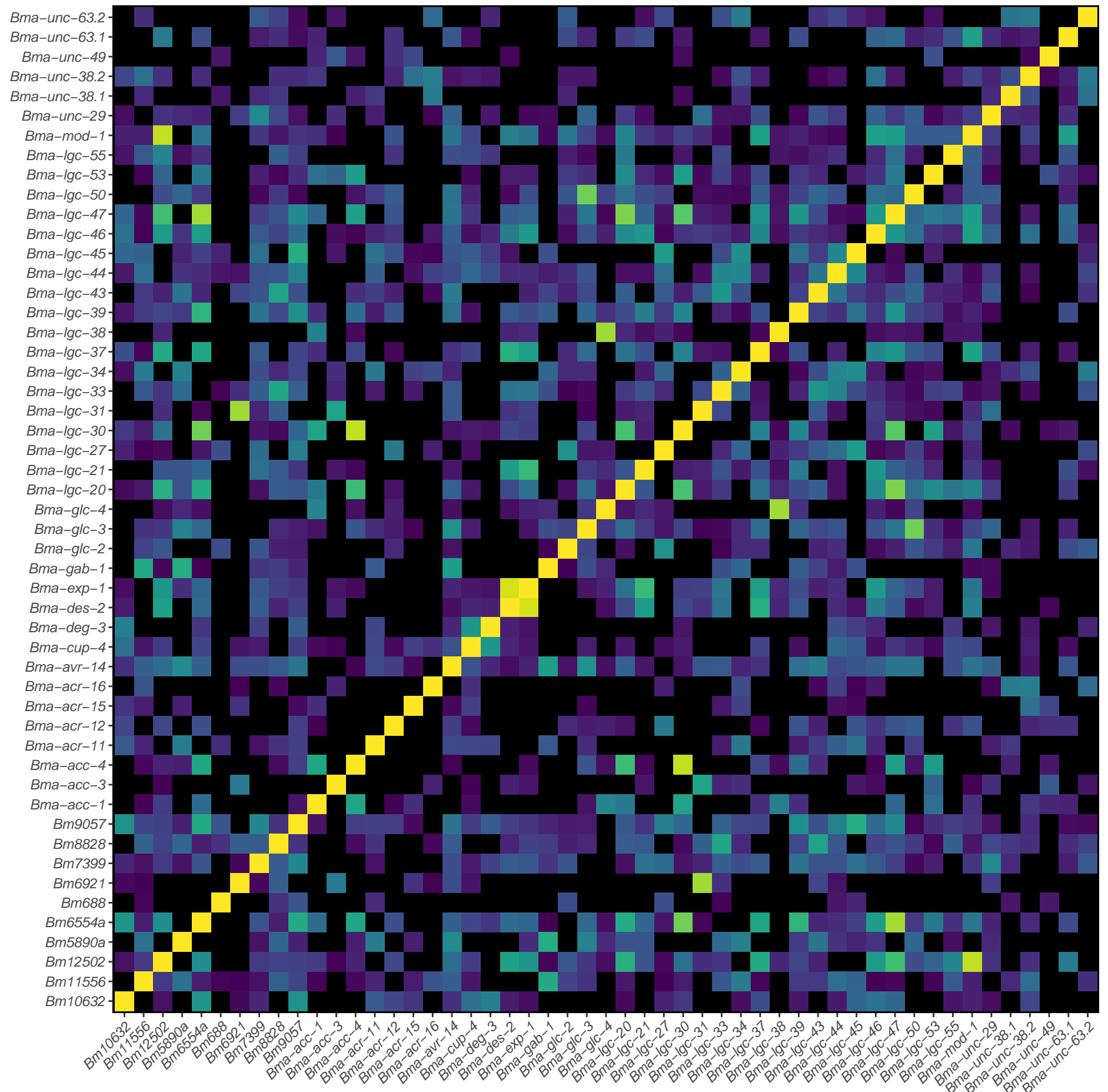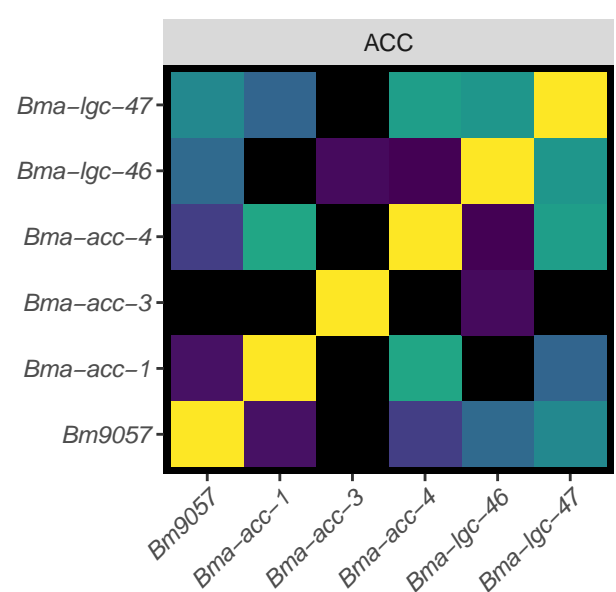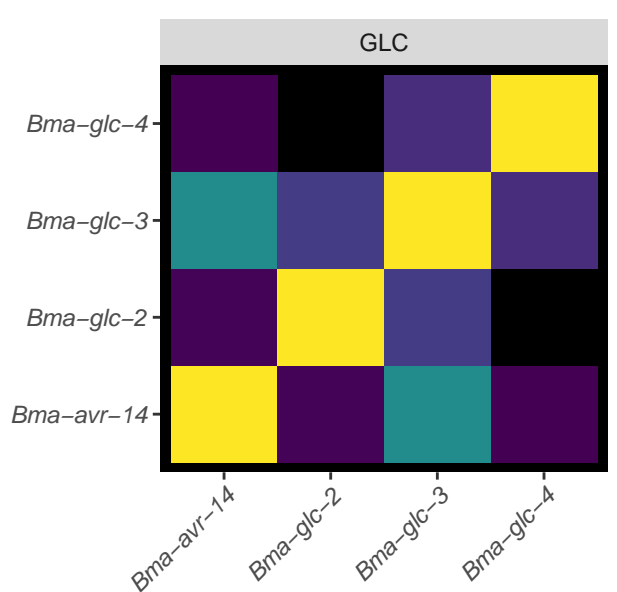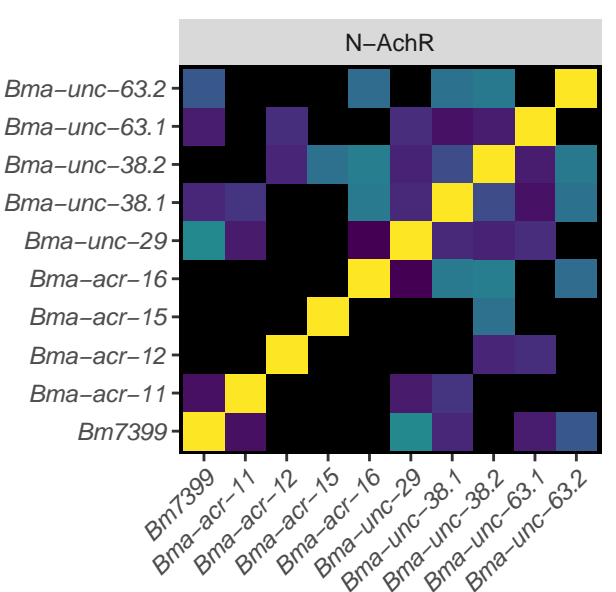

Supplement: S2 Fig — Pearson correlation coefficients were calculated from scale-normalized TPM values for the highest-quality RNA tomography replicate. (PDF) [file ppat.1010399.s008.pdf]

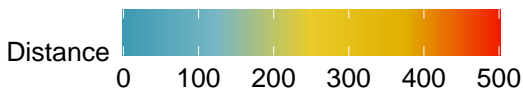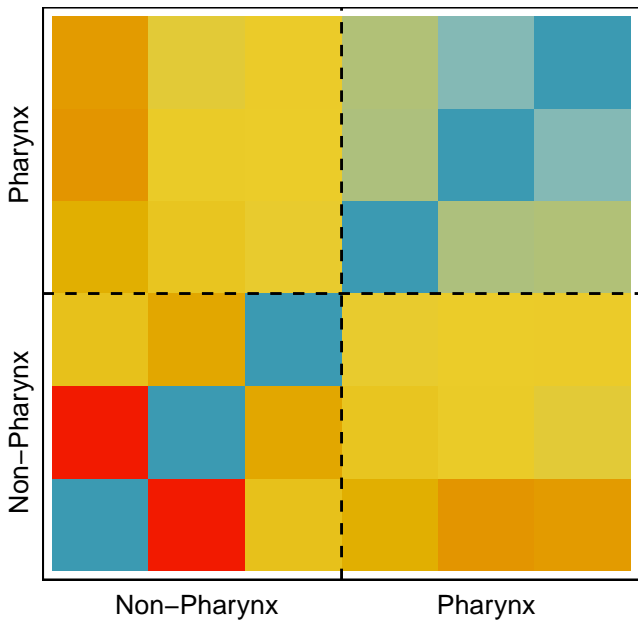

Supplement: S3 Fig — Samples are clustered based on euclidean distances of variance stabilizing transformed (vst) count data. (PDF) [file ppat.1010399.s009.pdf]
